# Cold ischemia or topical-ECMO for lung preservation: a randomized experimental study: Preservação pulmonar por isquemia fria ou ECMO-tópico: um estudo aleatório experimental

**DOI:** 10.1590/1516-3180.2014.1321594

**Published:** 2014-02-01

**Authors:** Alessandro Wasum Mariani, Israel Lopes Medeiros, Paulo Manuel Pêgo-Fernandes, Flavio Guimarães Fernandes, Fernando Do Vale Unterpertinguer, Lucas Matos Fernandes, Paulo Francisco Cardoso, Mauro Canzian, Fabio Biscegli Jatene

**Affiliations:** I MD, PhD. Attending Physician, Department of Thoracic and Cardiovascular Surgery, Instituto do Coração (InCor), Hospital das Clínicas (HC), Faculdade de Medicina da Universidade de São Paulo (FMUSP), São Paulo, Brazil; II MD, PhD. Attending Physician, Department of Thoracic Surgery, Hospital de Messejana, Fortaleza, Brazil; III MD, PhD. Full Professor of Thoracic Surgery, Instituto do Coração (InCor), Hospital das Clínicas (HC), Faculdade de Medicina da Universidade de São Paulo (FMUSP), São Paulo, Brazil; IV Medical Student, Faculdade de Medicina da Universidade de São Paulo (FMUSP), São Paulo, Brazil; V MD. Attending Physician, Department of Thoracic and Cardiovascular Surgery, Instituto do Coração (InCor), Hospital das Clínicas (HC), Faculdade de Medicina da Universidade de São Paulo (FMUSP), São Paulo, Brazil; VI MD, PhD. Professor, Faculdade de Medicina da Universidade de São Paulo (FMUSP), São Paulo, Brazil; VII MD, PhD. Attending Physician, Department of Pathology, Instituto do Coração (InCor), Hospital das Clínicas (HC), Faculdade de Medicina de Universidade de São Paulo (FMUSP), São Paulo, Brazil; VIII MD, PhD. Full Professor of Cardiovascular Surgery, Head of Thoracic and Cardiovascular Surgery Departament, Instituto do Coração (InCor), Hospital das Clínicas (HC), Faculdade de Medicina da Universidade de São Paulo (FMUSP), São Paulo, Brazil

**Keywords:** Organ preservation, Reperfusion injury, Lung transplantation, Transplantation, homologous, Thoracic surgery, Preservação de órgãos, Traumatismo por reperfusão, Transplante de pulmão, Transplante homólogo, Cirurgia torácica

## Abstract

**CONTEXT AND OBJECTIVE:**

Lung preservation remains a challenging issue for lung transplantation groups. Along with the development of *ex vivo* lung perfusion, a new preservation method known as topical-ECMO (extracorporal membrane oxygenation) has been proposed. The present study compared topical-ECMO with cold ischemia (CI) for lung preservation in an *ex vivo* experimental model.

**DESIGN AND SETTING:**

Randomized experimental study, conducted at a public medical school.

**METHOD:**

Fourteen human lungs were retrieved from seven brain-dead donors that were considered unsuitable for transplantation. The lung bloc was divided and each lung was randomized to be preserved by means of topical-ECMO or CI (4-7 °C) for eight hours. These lungs were then reconnected to an *ex vivo* perfusion system for functional evaluation. Lung biopsies were obtained at three times. The functional variables assessed were oxygenation capacity (OC) and pulmonary artery pressure (PAP); and the histological variables were lung injury score (LIS) and apoptotic cell count (ACC).

**RESULTS:**

: The mean OC was 468 mmHg (± 81.6) in the topical-ECMO group and 455.8 (± 54) for CI (P = 0.758). The median PAP was 140 mmHg (120-160) in the topical-ECMO group and 140 mmHg (140-150) for CI (P = 0.285). The mean LIS was 35.57 (± 4.5) in the topical-ECMO group and 33.86 (± 6.1) for CI (P = 0.367). The ACC was 25.00 (± 9.34) in the topical-ECMO group and 24.86 (± 10.374) for CI (P = 0.803).

**CONCLUSIONS:**

The present study showed that topical-ECMO was not superior to cold ischemia for up to eight hours of lung preservation.

## Abstract (pt)

**CONTEXTO E OBJETIVO:**

A preservação pulmonar permanece um desafio para os grupos transplantadores. Com o desenvolvimento da perfusão pulmonar *ex vivo*, foi proposto um novo método de preservação chamado de ECMO-tópico (oxigenação de membrana extracorpórea). O presente estudo compara ECMO-tópico com isquemia fria (IF) para preservação pulmonar em um modelo experimental *ex vivo*.

**TIPO DE ESTUDO E LOCAL:**

Estudo experimental randomizado, conduzido em uma faculdade de medicina pública.

**MÉTODO:**

Quatorze pulmões humanos foram retirados de sete doadores de morte cerebral considerados não aptos a transplante. O bloco pulmonar foi dividido e cada um foi aleatorizado para preservação por ECMO-tópico ou IF (4-7 °C) durante oito horas. Esses pulmões foram então re-conectados a um sistema de perfusão *ex vivo* para avaliação funcional. Biópsias pulmonares foram obtidas em três tempos. As variáveis funcionais avaliadas foram: capacidade de oxigenação (CO) e pressão de artéria pulmonar (PAP). As variáveis histológicas estudadas foram escore de lesão pulmonar (ELP) e contagem de células apoptóticas (CCA).

**RESULTADOS:**

A média da CO foi de 468 mmHg (± 81.6) no grupo ECMO-tópico e 455.8 (± 54) no grupo IF (P = 0,758); a PAP média foi de 140 mmHg (120-160) para ECMO-tópico e 140 mmHg (140-150) para IF (P = 0,285); o ELP médio foi 35,57 (± 4,5) no ECMO-tópico e 33,86 (± 6,1) no IF (P = 0,367). A CCA foi 25,00 (± 9,34) no grupo ECMO-tópico e 24,86 (± 10,374) no IF (P = 0,803).

**CONCLUSÕES:**

O presente estudo demonstrou que o ECMO-tópico não é superior a IF para oito horas de preservação pulmonar.

## INTRODUCTION

Lung transplantation has been established as a treatment option for end-stage lung disease.^1^ However, the low tolerance of lungs to ischemia is notorious and can lead to graft dysfunction that impacts on the recipient's outcome.^2^ Despite the variety of preservation techniques proposed, such as topical cooling,^3^ autoperfusion with extracorporeal circulation^4^ and donor core cooling,^5^ pulmonary artery flush perfusion with cold preservation solution has endured the test of time and has remained the most frequently used technique because of its practicality and effectiveness.^6^ Extracellular preservation solutions such as Perfadex (VitroLife AB, Gothenburg, Sweden) and Celsior (Sang Stat, Lyon, France) have been used frequently for lung preservation.^7^

Concomitantly to the introduction of *ex vivo* lung perfusion, a method of preservation named topical-ECMO (extracorporeal membrane oxygenation) has been proposed by Steen et al. It has been tested experimentally^8^ and used clinically.^9^ ^,^ ^10^ This method was designed to preserve the lungs after *ex vivo* assessment and consists of immersion of the lung in a semi-inflated state in Steen solution diluted in Perfadex inside the *ex vivo* box where the lungs were placed after reconditioning. Although topical-ECMO has been proposed and used clinically, the method has not been compared with cold ischemia after single-flush perfusion for preservation so far. 

## OBJECTIVE

The aim of this study was to evaluate whether topical-ECMO can provide better preservation quality than shown by regular cold ischemia, as methods for lung preservation.

## METHODS

This was a randomized experimental study approved by our university hospital's ethics committee (CAPPESQ 0212/08). Between December 2009 and August 2010, lungs retrieved from brain-dead donors that were refused for transplantation based on current clinical criteria were used in this study. The organs were included in the protocol if all efforts to recover the lungs failed. Written consent was obtained from family members of the donors to permit the use of the organs in this study. The lungs were perfused through the pulmonary artery with cold Perfadex (Vitrolife, Gothenburg, Sweden) and harvested in the usual fashion at the time of multiorgan retrieval. The organs were immersed in cold Perfadex, stored in a cooler and transported to our laboratory.

**Figure 1:**
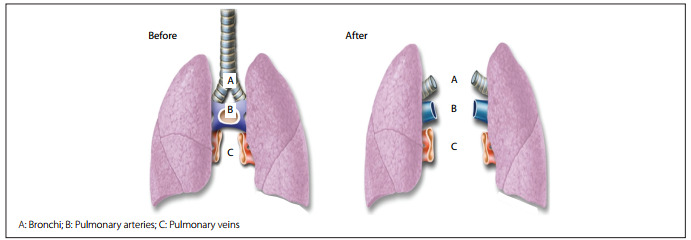
Preparation of the double-lung block before (left) and after (right) separation of the grafts.

Upon arrival, the lung block was dissected out, and the left and right lungs were separated by sectioning the left atrium, main pulmonary artery and the tracheal carina (Figure 1). Separation of the lungs allowed each lung to be subjected to a different forms of preservation. The lungs were then randomly assigned for topical-ECMO or cold ischemia (CI). The randomization was done by means of numbered envelopes containing computer-generated random sequences of numbers.

**Figure 2:**
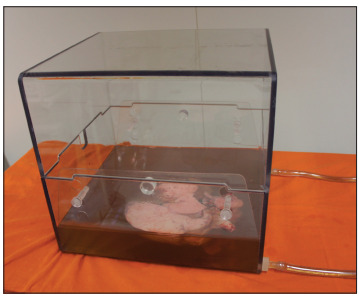
Containment box for topical-ECMO, with lung immersed in Steen Solution.

Topical-ECMO was carried out by means of complete lung immersion in solution (Steen Solution; VitroLife AB, Gothenburg, Sweden) within a containment box (VitroLife AB, Gothenburg, Sweden) (Figure 2). The solution was continuously circulated through this box by means of a centrifugal pump (Braile Biomedica, Sгo Josй do Rio Preto, Brazil) with a flow of 4 l/min and through a membrane oxygenator (Braile Biomedica, Sгo Josй do Rio Preto, Brazil) receiving oxygen at 5 l/min in order to achieve oxygenation. The average temperature was maintained by means of a heat exchanger with a target of 8 °C. The lung temperature was monitored continuously using a thermometer placed inside the pulmonary vein. 

CI lungs were stored in a bag containing Perfadex solution and were immersed in a second plastic bag containing saline solution at 4 °C. The bags were then stored in a refrigerator at a temperature of 4-7 °C.

**Figure 3:**
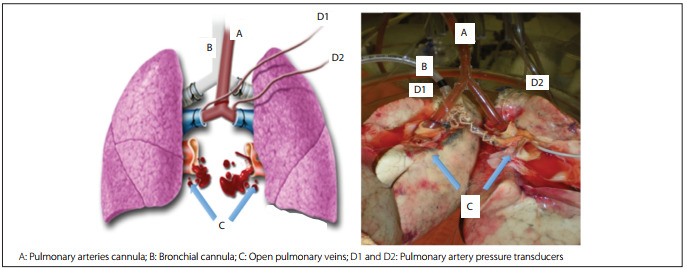
Drawing (left) and photograph (right) of the ex vivo lung system with reconnection used in the experiments.

After eight hours of preservation, the left and right lungs were reconnected in parallel, by means of two Y-shaped cannulae: one in the trachea and the other in the pulmonary artery. The pulmonary veins remained separated. This reconnection technique made it possible for the topical-ECMO and CI lungs to undergo reperfusion and ventilation in the *ex vivo* perfusion system simultaneously, with the same reperfusion solution and exactly the same ventilation parameters. Since the pulmonary veins remained independent, it enabled sampling for blood gas analysis separately (Figure 3).

The system used during the reperfusion phase consisted of the XVIVO box (VitroLife AB, Gothenburg, Sweden), attached to the centrifugal pump, heat exchanger, membrane oxygenator and a venous reservoir (Braile Biomedica, Sгo Josй do Rio Preto, Brazil), as we previously described.^11^ The Y cannula for the pulmonary arteries had two separate probes connected to pressure transducers that allowed continuous and independent monitoring of pulmonary artery pressure. The right and left lung pulmonary vein effluents were collected directly into the XVIVO box, where they were mixed and drained into the venous reservoir by gravity. We elected to keep the atria open in order to facilitate assembly of the system, by eliminating the need for special atrial cannulae. This is feasible during short-term perfusion, whereas in cases requiring long-term perfusion, a closed system is preferred so as to avoid pulmonary edema.^12^ The system was filled with 1,500 ml of Steen Solution (VitroLife AB, Gothenburg, Sweden), and we chose to use a bloodless cellular solution. The pH was adjusted between 7.35-7.45 by adding trometamol (Addex-THAM; Fresenius-Kabi AB, Uppsala, Sweden). A maximum perfusion flow of 40% of the estimated cardiac output (CO) was utilized, and this was calculated using a formula based on the size of the donor (CO = 3 * body surface area, assuming target cardiac index = 3). This flow was sufficient to assess the lungs in the system and low enough to avoid pulmonary edema formation.

A steady state was usually reached after 60 minutes of perfusion, and perfusate gases were collected and hemodynamics were recorded at this time. The variables assessed were pulmonary arterial partial oxygen pressure (PvO_2_), pulmonary partial oxygen pressure of the effluent from the pulmonary veins (PaO_2_) and partial carbon dioxide (PaCO_2_), pulmonary artery pressure (PAP) and pulmonary vascular resistance (PVR) [PVR = PAP/pulmonary artery flow * 80 (dynes/sec/cm^5^)]. 

**Figure 4:**
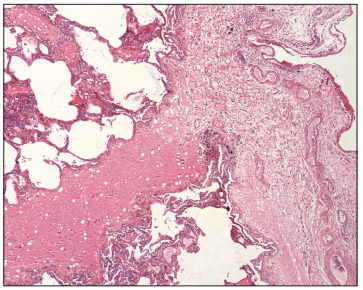
Histological appearance of the lung after ex vivo perfusion (original magnification 100 x; hematoxylin-eosin).

Lung tissue samples from the middle lobe and ligulae were collected before harvesting, after the topical-ECMO or cold ischemia period and after *ex vivo* lung perfusion (EVLP). The samples were fixed in 10% buffered formalin for 24 hours, embedded in paraffin, sectioned at thicknesses of 5 mm and stained with hematoxylin and eosin (Figure 4). For all cases, semiquantitative scoring was performed by an experienced lung pathologist using the following histology parameters: interstitial edema, intra-alveolar edema, arteriolar thickening, vascular thrombosis, intra-alveolar hemorrhage, intra-alveolar fibrin deposition, necrosis, inflammatory cell infiltrate, pleural infiltrate, peribronchiolar inflammatory reaction, organizing pneumonia, peribronchiolar fibrosis, fibroblast foci, peribronchiolar muscular hypertrophy, pleural plaques, interstitial fibrosis, alveolar lining cell hyperplasia, alveolar macrophages, pigmented macrophages, denudated bronchiolar epithelium, vasculitis and emphysema. The severity of these findings was determined using a four-grade scale: absent = 0; minimal = 1; moderate = 2; and intense = 3. The sum of each parameter resulted in the Lung Injury Score (LIS), with values ranging from 0 to 66.^13^

Apoptosis was assessed by means of *in situ* terminal deoxynucleotidyl transferase (TdT)-mediated deoxyuridine triphosphate nick end labeling (TUNEL), using the In Situ Cell Death Detection Kit (Roche, Basel, Switzerland). The TUNEL method is based on the enzymatic ability of TdT to catalyze a template-independent addition of deoxyribonucleotide triphosphate to the 3=-OH ends of double- or single-stranded DNA. The sections were deparaffinized and rehydrated. Protein digestion was done by applying proteinase K to the slides for 15 minutes at room temperature, which was followed by four washes in distilled water for two minutes each. Equilibration buffer was applied to the sections, which were then incubated in a humidified chamber for three minutes. Following this, the sections were incubated with TdT in a humidified chamber at 37 °C for 1 hour. Fluorescein-labeled antidigoxigenin antibody was applied to the sections, and they were incubated in a humidified chamber for 30 minutes at room temperature. The sections were then washed with phosphate-buffered saline.

**Figure 5:**
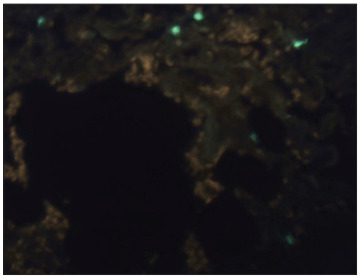
Photomicrograph demonstrating apoptotic cells (green) under a fluorescence optical microscope.

Immediately after completing the protocol, the slides were viewed using a fluorescence microscope and photographed using a charge-coupled device digital camera with a 590-nm emission filter. To localize apoptotic cells, randomly selected specimens of lung tissue were photographed under the fluorescence optical microscope at high magnification (original magnification 400 x). Apoptotic cells appeared as bright green (Figure 5). Counts were obtained from five randomly chosen fields per slide, representing a total area of 0.1 mm^2^. An independent, blinded examiner performed the cell counting.

Immunohistochemical analysis was performed using the polyclonal rabbit antibody for CD3 (1:100, Abcam, Cambridge, United Kingdom). The sections were deparaffinized and a 0.3% hydrogen peroxide solution was applied for 35 min to inhibit endogenous peroxidase activity. Antigen retrieval was performed using citrate solution for 45 minutes. The sections were incubated with the primary antibody overnight at 48 °C. The streptavidin-biotin complex (LSAB+; DakoCytomation, Carpinteria, CA, United States) was used as the secondary antibody and 3,3-diaminobenzidine (DAB) (Sigma Chemical Co, St. Louis, MO, United States) was used as the chromogen. The sections were counterstained using Harris hematoxylin. For negative controls, the primary antibody was replaced with phosphate-buffered saline (PBS). The inflammation index was determined semiquantitatively based on the presence of inflammatory cells in lung tissue, using a three-grade scale: minimal inflammation = 1, moderate inflammation = 2 and marked inflammation = 3.

All histological and immunohistochemical parameters were evaluated and measured by a pulmonary pathologist who was blinded to any information regarding the cases.

Lungs were weighed immediately after harvesting, at the end of the preservation time and after reperfusion. They were also subsequently dried out for 48 hours at 70 °C and weighed again in order to obtain the wet/dry weight ratio.

The normality tests applied were Kolmogorov-Smirnov and Shapiro-Wilk. To compare functional and histological data from before to after the procedure, Student's paired-samples test was performed. The Wilcoxon signed rank test was used for variables that were not normally distributed. Repeated-measurement ANOVA was used to analyze the differences in measurements that evolved over time in pairs of groups. For qualitative variables in 2 x 2 tables, we used the chi-square test, or Fisher's exact test when the expected value was less than five. In tables larger than 2 x 2, the likelihood function was used because the expected value was less than five in all cases. The results were expressed as the mean and standard error of the mean, or as the median and interquartile range for variables that were not normally distributed. The statistical analyses were performed using the SPSS (Statistical Package for the Social Sciences) 18.0 software (SPSS Inc, Chicago, IL) with a confidence interval of 95% and a significance level of 0.05.

## RESULTS

Seven sets of lungs were assessed. The demographics are summarized in Table 1. During reperfusion, the oxygenation capacity was estimated from the PaO_2 _measured in the effluent perfusate from the pulmonary veins of the lungs. For the lungs preserved by means of topical-ECMO and CI, respectively, the capacities were 468 ± 81.6 mmHg and 455.86 ± 54 mmHg, with no statistically significant differences between the groups (P = 0.758). The mean partial oxygen pressure of the deoxygenated perfusate inflow during EVLP (PvO_2_) was 91.94 ± 24.4 mmHg. The mean arterial PaCO_2_ in lungs preserved by means of topical-ECMO and CI were 17.45 ± 3.6 mmHg and 17.02 ± 3.1 mmHg respectively, with no statistically significant differences between the groups (P = 0.617).

**Table 1:**
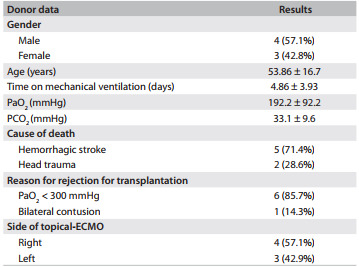
Demographics and clinical characteristics of the donors

ECMO = extracorporal membrane oxygenation.

Hemodynamics showed that the median PAP in topical-ECMO lungs was 140 mmHg (120-160) and in CI lungs, 140 mmHg (140-150); P = 0.285. The median PVR also showed no statistically significant differences between the groups: topical-ECMO = 459 dynes/sec/cm^5^ and CI = 474.50 dynes/sec/cm^5^; P = 0.285.

**Figure 6:**
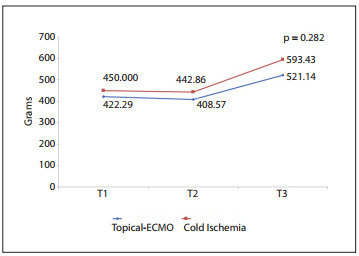
Lung weight gain between groups over time comparing topial ECMO (extracorporal membrane oxygenation) and CI (cold ischemia).

**Figure 7:**
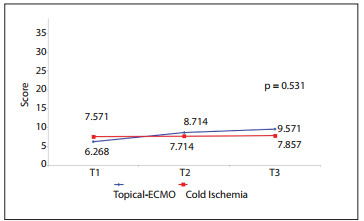
Comparison of lung injury scores between groups over time comparing topical ECMO (extracorporal membrane oxygenation) and CI (cold ischemia).

**Figure 8:**
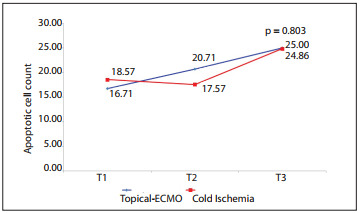
Comparison of apoptotic cell counts between groups over time comparing topical ECMO (extracorporal membrane oxygenation) and CI (cold ischemia).

The mean weight changes of the lungs between the groups over time (pre-ischemic, post-ischemic and post-reperfusion times) did not show any significant differences, as depicted in Figure 6. The wet-to-dry ratios in the topical-ECMO and CI groups were 2.77 ± 0.93 and 3.21 ± 1.85, respectively, with no statistically significant difference (P = 0.358). The lung histology, studied by means of LIS and the apoptotic cell count (ACC), did not show any significant differences between the groups over time (LIS P = 0.531 and ACC P = 0.803), as shown in Figures 7 and 8.

The degree of tissue inflammation was assessed by detecting the presence of inflammatory cells. Infiltration of CD3+ T-lymphocytes into the lung tissue was evaluated at three times and did not significantly differ between the groups, as shown in Table 2.

**Table 2:**
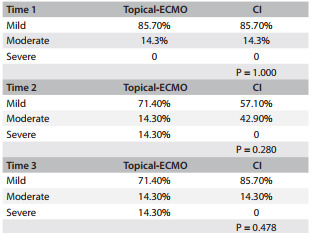
Degree of CD3 T-lymphocyte infiltration into lung tissue at three times

ECMO = extracorporal membrane oxygenation; CI = cold ischemia

## DISCUSSION

Our experiments showed that there were no differences between topical-ECMO and CI in terms of preservation quality, hemodynamic performance and histology of the lungs assessed.

Although topical-ECMO was created and has been used as a method for lung preservation at Lund University in Sweden, it has not been tested for efficacy or safety. In the first paper in which this method appears, 12 pigs were used as non-heartbeat donors and lung function was assessed *ex vivo* after harvesting. After the *ex vivo* evaluations had been completed, the lungs were cooled and preserved using topical-ECMO at a temperature of 12 °C, until transplantation.^8^ The next paper described the first human case that received a lung after *ex vivo* evaluation. After perfusion, the left lung was stored using topical-ECMO at 8 °C and remained there for nine hours and 30 minutes, until transplantation.^9^

In 2009, the same group published six human cases of transplantation after *ex vivo* lung perfusion. Once again, after the *ex vivo* perfusion had been completed, the lungs were cooled down and stored using topical-ECMO until transplantation. The mean duration of use of topical-ECMO was five hours and 53 minutes for the first implanted lung and 8 hours and 46 minutes for the second lung.^10^ Despite the good results in all these papers, it cannot be affirmed whether the use of topical-ECMO interfered with lung preservation or with lung function, either positively or negatively.

Since the time when the topical-ECMO method was designed, this is the first comparative study evaluating it against any other preservation strategy. Our use of rejected human lungs in an experimental study was motivated by the aim of testing the method in a realistic situation that would also be more representative of the clinical lung transplantation scenario. The study was designed such that it would optimize the use of rejected donor lungs and, likewise, would reduce variability by using both lungs from the same donor, i.e. one lung for each of two different preservation techniques.

The difference between the topical-ECMO described by Steen et al. and the one used here was the solution. While the Swedish group^8^ ^-^ ^10^ used a mixture of Steen Solution, Perfadex and a variable quantity of red blood cells in order to reach a hematocrit level of around 5%, pure Steen Solution was used in the present work. 

The concept of splitting the double-lung block and sharing both the airway and the inflow perfusion connections had been tested previously in a pilot study in our laboratory and was proven to be feasible without significant functional deterioration of the lungs.^14^

The oxygenation capacity during the *ex vivo* perfusion can be considered to be the most important parameter for functional assessment, since it reflects both gas exchange and graft performance. Previous studies have shown that PaO_2_ values correspond more reliably to the quality of lung preservation than do other parameters such as those from pathology or radiology.^15^ In the present study, the absence of significant differences between the groups demonstrates that oxygen capacity and CO_2_ clearance were similar in both groups.

The parameter of weight variation has been used experimentally as a reliable measurement of pulmonary edema in the setting of lung preservation. The process of ischemia and reperfusion results in increased vascular permeability and disruption of the alveolar-capillary barrier, which ultimately causes water extravasation. The amount of edema can be therefore considered to be inversely proportional to the quality of preservation.^16^ Despite the usefulness of weight variation in determining edema, its accuracy is debatable since it is influenced by other factors such as alveolar hemorrhage, which also renders the method less accurate for detection of milder degrees of edema. In our study, the weight gain after reperfusion was similar in both groups and possibly reflected the degree of edema inherent to the *ex vivo* reperfusion, which may become an adverse factor during longer reperfusions, as described previously.^12^ On the other hand, the wet-to-dry ratio has been found to be a more reliable measurement of the amount of water in the specimens at the end of reperfusion.^17^ Therefore, the higher the value is, the greater the edema will be. In our study, the wet-to-dry ratio was similar in both groups, thus suggesting that similar amounts of edema were present regardless of the preservation strategy used.

There are two major limitations regarding the histological changes found in the lungs: the degree of the histological changes does not show any linear correlation with the functional outcome; and the presence of pulmonary parenchymal lesions before harvesting, induced by pro-inflammatory agents secondary to brain death, may be great enough to cause profound changes to the pulmonary tissue.^18^ Nevertheless, histological evaluation remains a powerful indicator of the quality of lung preservation.^19^ The injury score used in this study (ELP) was similar between the groups, both after the period of preservation and after reperfusion. This finding shows that the two preservation methods yielded comparable preservation quality.

ACC performed by means of an immunohistochemical technique (TUNEL) has been studied in human lung transplant cases.^20^ Fischer et al. found that there was a significant increase in the number of apoptotic cells two hours after graft reperfusion.^21^ These studies demonstrated that there was a significant correlation between the percentage of necrotic cells and deterioration of graft function, as estimated by means of PaO_2_ after implantation. In our study, the ACC was equivalent in the two preservation techniques.

Immunohistochemistry was used to stain CD3+ cells. The lymphocytic infiltrate was quantified in order to assess the magnitude of the inflammatory infiltrate, and we found no differences between the groups. This was indicative that the two preservation methods were comparable with regard to inflammation. On the other hand, the shorter period of *ex vivo* reperfusion in our study (eight hours) may have played a role in these findings. Cypel et al. used a similar method for comparing lung preservation by means of an *ex vivo* reperfusion system, with cold ischemia for twelve hours. Their study showed that *ex vivo* reperfusion presented lower levels of lymphocyte infiltrate, thus indicating lower intensity of inflammation and therefore better lung preservation.^22^

The present study has several limitations. The small number of cases and the presence of lung injury prior to harvesting, plus the fact that no lungs were transplanted and reperfused, are among the most prominent of these. Because of the great difficulty in obtaining human lung donors for experimental research (small numbers of cases, non-acceptance by the relatives and logistic difficulties), we had to base our sample on opportunity sampling (with no prior sample calculation). The high variability between cases of lung donors that may exist in such investigations was the reason for developing the "split lung block technique".^14^ Our attempt to minimize bias by pairing each set of lungs from the same donor may have mitigated some of the factors, but many aspects remain unclear and will require future studies. The small number of cases makes it impossible for us to extrapolate the results from this study to the general population.

The implications from this study for lung transplantation practice lie in the fact that the topical-ECMO technique, which is more complex and expensive, does not seem to bring any benefits regarding lung preservation. Within the field of research, this work may contribute through providing additional data on the use of *ex vivo* lung perfusion systems, in ischemia-reperfusion studies.

## CONCLUSION

The results show that topical-ECMO does not seem to improve lung preservation, compared with cold ischemia, for up to eight hours of preservation.
